# Pain Perception Threshold in Young High-Altitude Natives After Acute Exposure to Severe Hypoxic Conditions

**DOI:** 10.3390/oxygen5010001

**Published:** 2025-01-13

**Authors:** Kely Melina Vilca-Coaquira, Angel Gabriel Calisaya-Huacasi, Jeancarlo Tejada-Flores, Henry Oscar Tintaya-Ramos, Mariela Mercedes Quispe-Trujillo, Solanyela Anny Quispe-Humpiri, Rossela Alejandra Rojas-Chambilla, Gilberto Félix Peña-Vicuña, Alberto Salazar Granara, Luis F. Lens Sardón, Alcides Flores-Paredes, Moua Yang, Ginés Viscor, Ivan Hancco Zirena

**Affiliations:** 1 Facultad de Medicina Humana, Universidad Nacional del Altiplano, Puno 21000, Peru; 2 ACEM (Asociación Científica de Estudiantes de Medicina), UNA, Puno 21000, Peru; 3 Centro de Investigación en Medicina de Altura (CIMA), Facultad de Medicina Humana, Universidad de San Martín de Porres, Lima 15001, Peru; 4 Facultad de Educación, Escuela Profesional de Educación Física, Universidad Nacional del Altiplano, Puno 21001, Peru; 5 Bloodworks Northwest Research Institute, Seattle, WA 98102, USA; 6 Division of Hematology and Oncology, Department of Medicine, University of Washington School of Medicine, Seattle, WA 98195, USA; 7 Sección de Fisiologia, Departament de Biologia Cellular, Fisiologia i Immunologia, Facultat de Biologia, Universitat de Barcelona, E-08028 Barcelona, Spain; 8 Division of Hemostasis and Thrombosis, Beth Israel Deaconess Medical Center, Harvard Medical School, Boston, MA 02115, USA

**Keywords:** pain tolerance, hypoxia, pain threshold, tourniquet test, ischemic pain

## Abstract

Previous studies indicate that individuals at high altitudes have a lower pain threshold than those living at sea level. This study evaluates the differences in pain perception among young people living at an altitude of 3800 m and after acute exposure to a severe hypoxic environment at more than 5100 m. Fourteen people (BMI of 22.6 ± 1.2 and age of 23.3 ± 1.9 years) residing in the city of Puno (3825 m) participated in an ascent to the Populated Center of La Rinconada (>5100 m). The unilateral ischemia pain provocation test was used, applying pressure with a manual sphygmomanometer to generate transient ischemia in the arm while the patient opens and closes their hand. Onset, peak, and resolution times of pain, heart rate, and oxygen saturation were recorded. At their residence altitude of 3828 m, the mean hemoglobin was 16.16 ± 2.29, while at 5100 m, mean hemoglobin increased to 17.57 ± 1.74. The average time to pain onset in the right arm was 30.43 s ± 14.15 at 3828 m, whereas at 5100 m above sea level, the pain perception was at 31.00 s ± 19.01. At 3828 m, the average time until pain sensation in the left arm was 19.93 s ± 9.44 and increased to 23.07 s ± 10.83 at 5100 m. During exposure to a severe hypoxic environment, the pain perception threshold was similar between 3828 m and 5100 m above sea level.

## 1. Introduction

Pain is one of the most frequent causes of medical consultation, with the most common cause being diseases of the musculoskeletal system [1]. It represents a primitive stimulus in humans and its importance in biological function is to provide a warning or alarm signal in the event of an injury, illness, or other noxious phenomenon [2]. This signal is originated by nociceptive stimuli that are detected by free nerve endings and is transported by type Aδ and C neurons, which may include myelination and are both slow-conducting fibers [3].

Pain generates a deterioration in the quality of life of the people who chronically suffer from it. Despite being an important and frequent symptom, an adequate evaluation of pain concerning its intensity, functional impact, and possible etiology is needed in order to decrease its clinical burden [4]. Thus, a methodology for a holistic assessment of pain can improve the quality of life in patients, as measured by changes in the quality of sleep as one potential output [5]. Pain can also have an emotional and social impact, altering daily life, and can impact other pathologies. When evaluated systematically, pain symptoms could allow for early detection and prevention of many diseases.

In high altitude regions, the decrease in the partial pressure of oxygen in inspired air (PIO_2_) produces a decrease in the ability of tissue cells to receive and use oxygen effectively. This state of hypoxia occurs at many different levels, both physiological and biochemical [6]. Furthermore, at high altitudes, a difference in altitude of about 1000 m has a much greater physiological impact than if the same ascent were made from sea level. This is due to the non-linear relationship between geographic altitude and barometric pressure, and, therefore, the availability of oxygen. Previous studies showed that during the early stages of adaptation to high altitude, changes in sensory perception occur [7]. An immediate decrease in the threshold of 25–40% has been observed in various senses, including touch, carbon dioxide sensitivity, color and light perception, taste, and smell, when subjects ascend to an altitude of more than 3400 m. This apparent decrease in sensation is reversed by supplemental oxygen administration [8]. However, sensory thresholds, and particularly pain threshold, can be strongly biased by ethnocultural, attentional, motivational, and genetic factors. A previous study on the effect of pain among people living at two different altitudes (sea level vs. almost 1900 m) reported that the pressure pain threshold is higher (i.e., pain sensitivity is lower) in highlanders compared to lowlanders, and that attitudes towards imaginary painful situations are lower in highland populations compared to lowland populations probably as a long-term effect of moderate altitude exposure [7]. On the other hand, during an expedition to the Bhrikuti Peak in the Himalayas (6460 m), a decrease in the pain threshold was observed in comparison with the control group at sea level [9].

Although the mechanisms of how the human body adapts to high altitude are well-understood, the specific impact on pain perception, especially in severe hypoxia at very high-altitude locations (e.g., La Rinconada) remains largely unknown. La Rinconada is a city located at an altitude of more than 5100 m and is considered the highest place of permanent residence in the world [10,11]. An understanding of how hypoxia impacts pain perception would improve theragnostic approaches to patients suffering from pain regardless of their altitude of residence. In this study, we hypothesize that pain perception could be different in the high-altitude environment of La Rinconada compared to a lower-altitude environment. In this study, we evaluated pain perception in a group of young Andean native subjects who live at around 3800 m and the changes that occur in pain perception when they are acutely exposed to a severe hypoxic environment of more than 5100 m of natural geographic altitude.

## 2. Materials and Methods

### Study population:

Fourteen subjects (7 males and 7 females; BMI of 22.6 ± 1.2 and age of 23.3 ± 1.9 years) residing in Puno at 3825 m above sea level (Pb = 65.1 kPa) participated in this study. Volunteers who agreed to undergo the different evaluations ascended to the Populated Center of La Rinconada, located at an altitude of more than 5100 m (Pb = 58.9 kPa). All subjects were healthy with no history of diseases or conditions that could alter the perception of pain.

### Procedure:

The initial evaluation of the subjects was carried out in Puno and a second evaluation was performed in La Rinconada. The following data were recorded: age, sex, weight, height, and body mass index. Systolic blood pressure (SBP), diastolic blood pressure (DBP), and heart rate (HR) were registered using a Ri-Champion digital blood pressure monitor (Rudolf Riester GmbH, Jungingen, Germany) with a measuring range of 30 to 280 mm Hg for blood pressure and a range of 40 to 200 beats per minute for heart rate. SatO_2_ was measured with a NELLCOR^®^ OXIMAX^®^ N-65 pulse oximeter (Digicare Biomedical Technology Inc., Boynton Beach, FL, USA) with a saturation resolution of 1% and a heart rate range of 30 to 235 beats per minute. Capillary blood samples were obtained from the pad of the finger of each participant. A puncture was performed with a sterile lancet to absorb a drop of blood in a microcuvette. The hemoglobin was measured with a Hb-201 hemoglobinometer (Hemocue, Ängelholm, Sweden), using the azidimethemoglobin method within a measurement range of 0 to 25.6 g/dL. Hematocrit (Hct) was measured in a HemataStat II microcentrifuge (EKF Diagnostics, Penarth, UK) on blood samples obtained by a fingertip puncture.

### Measurement of pain tolerance (Tourniquet ischemic test):

Ischemic pain is elicited by the ischemic handgrip exercise (20 times) of the subject after a tourniquet is inflated around the upper arm. The quality of sensation is dull-aching or stinging muscular pain, which closely resembles most types of pathologic pain, but increases progressively after cessation of squeezing. Test performance is measured in terms of elapsed time between cessation of squeezing and report of slight (threshold) and unbearable (tolerance) pain. Muscular pain from ischemic contractions, which is due to transient stimulation of peripheral nociceptors [12], is based on the algogenic actions of protons [13]. The ischemia procedure was performed as previously described [14]. In brief, the patient is in a sitting position with the arm resting on a table. The arm remains at the same level as the heart to place the manual blood pressure monitor with the lower edge 2 cm above the bend of the elbow. The cuff was insufflated up to 200 mmHg. Then the patient opens and closes their hand rhythmically. A specific cadence for hand open-close was not established with a metronome although it was similar between subjects at around 1 Hz. The evaluator records, with a stopwatch, the times of pain onset, when the pain becomes unbearable, and when the pain disappears. These timing data also allow us to calculate the exasperation time (exasperation = unbearable − onset) and the resolution time (resolution = disappearance − unbearable). Additionally, during this procedure, both heart rate and oxygen saturation were measured. This test was performed in random order on both arms as the original protocol suggests [12].

### Statistical analysis:

The data presented are the mean values and standard deviation. The normality distribution of datasets was determined with the Shapiro–Wilk test, which resulted in all variables showing a normal distribution. Simple linear regression was calculated between some pairs of variables. Data processing was performed using the IBM SPSS statistical package version 26.

### Ethical aspects:

Before the study, each participant received detailed information about the investigation’s procedures and objectives. All the subjects signed an informed consent form before participating in the study. The study was approved by the Ethics Committee of the Universidad de San Martin de Porres with FWA International Registry for the Protection of Human Subjects No.00015320, IRB No. 00003251.

## 3. Results

Fourteen subjects with similar characteristics were evaluated and divided into two groups (*n* = 7) according to gender. The BMI of the subjects was 22.6 ± 1.2 and the average age of the participants was 23.3 ± 1.9 years.

**Figure 1.**
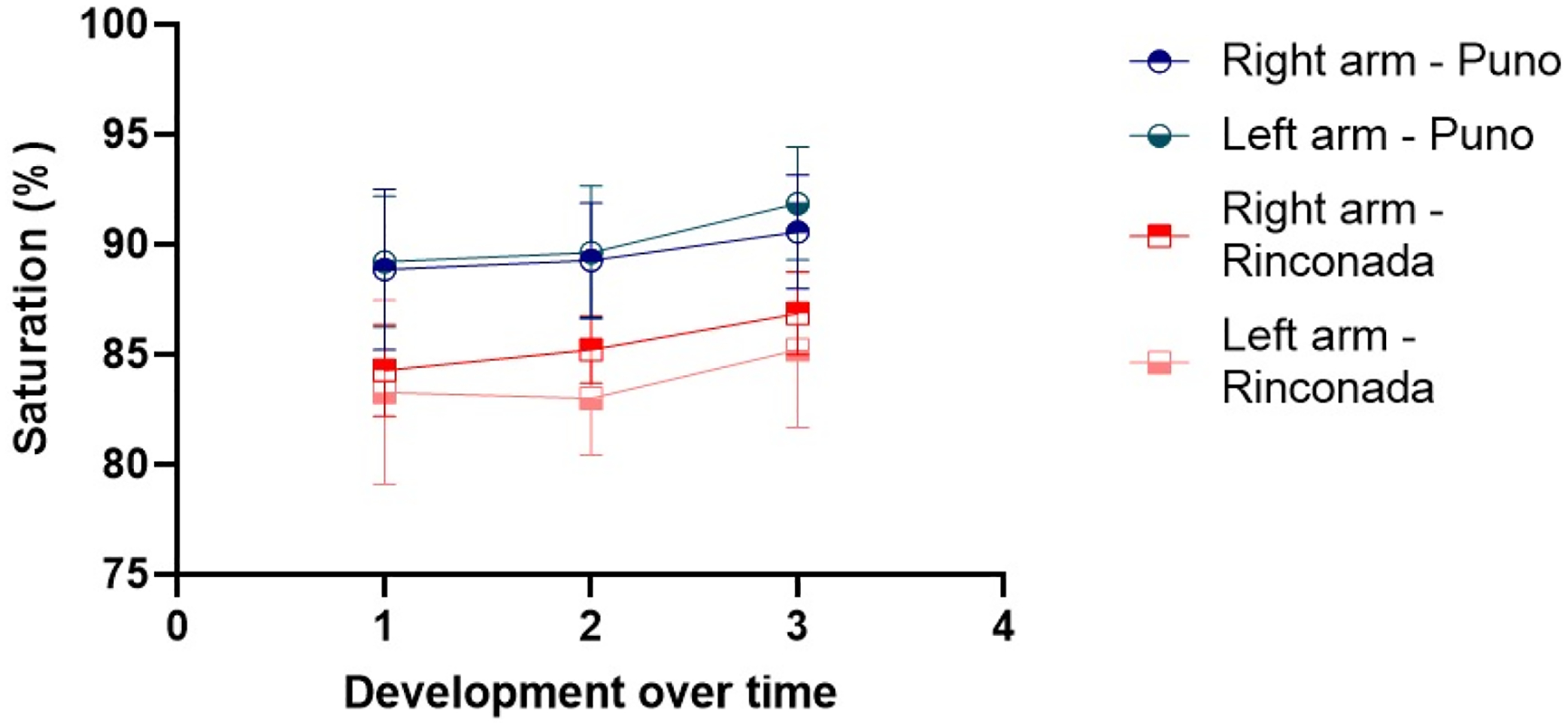
Changes in oxygen saturation during the pain threshold test at the two altitudes on the right and left arms. Labels for x-axis are time of first pain (1), unbearable pain (2), and no pain (3).

**Figure 2.**
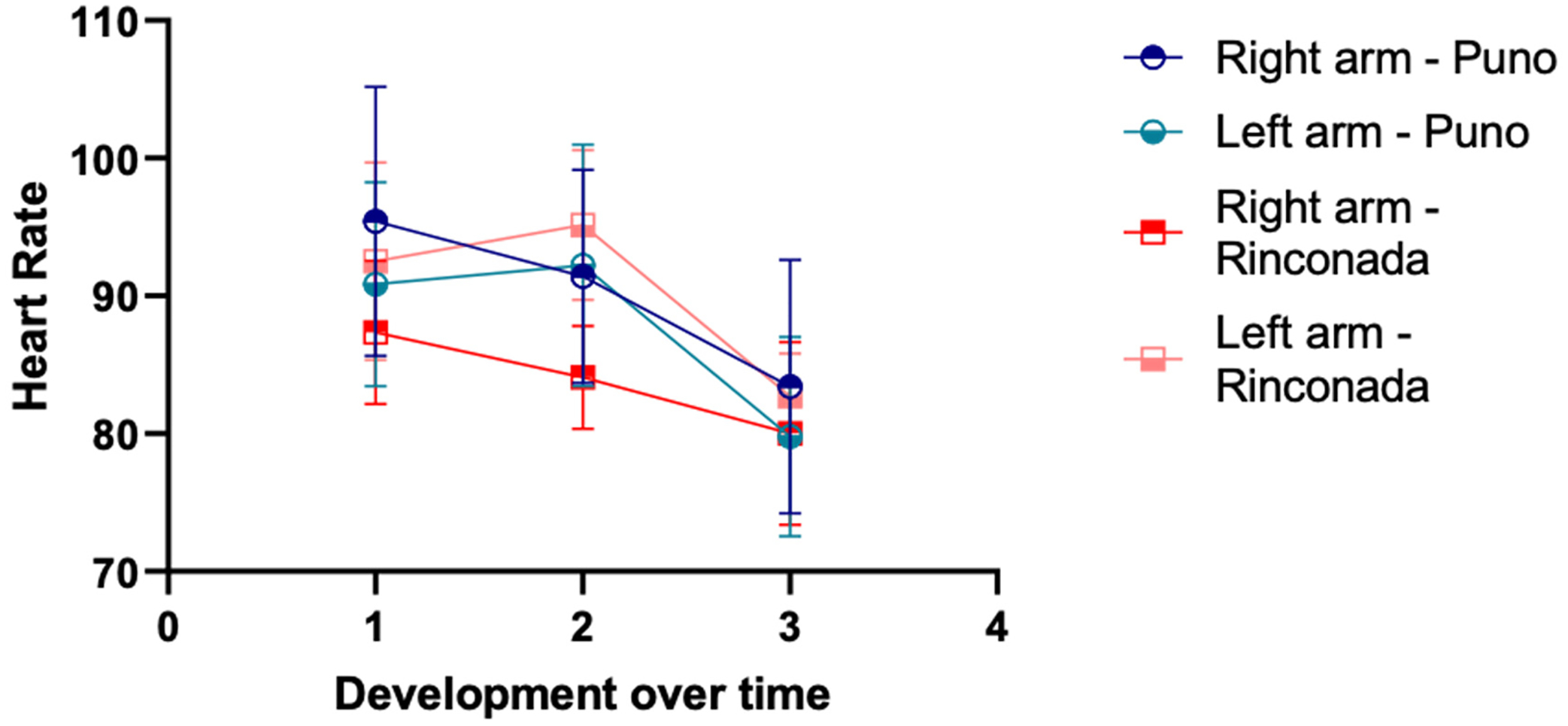
Heart rate changes during the pain threshold test at the two altitudes on the right and left arms. Labels for x-axis are time of first pain (1), unbearable pain (2), and no pain (3).

As expected, 24 h after arrival at an altitude of 5100 m, a significant increase in Hb (about 1 g/dL) and Hct (approximately 4%) were evidenced. Also, statistically significant increases in SBP and DBP and a decrease in SatO_2_ were observed (Table 1). Concerning the pain perception in the right arm, the onset tended to be slightly sooner at 5100 m for approximately a half second (non-significant difference), and the pain sensitivity dynamics were similar between the two altitudes (Table 2). In the left arm, we found similar results, although the time of pain onset tended to be longer at 5100 m (non-significant difference), whereas the exasperation time was significantly shorter at 5100 m (Table 3). Strikingly, the resolution time was significantly longer at the higher altitude for the two arms. Differences in SatO_2_ are observed as a function of altitude with lower levels at 5100 m (Figure 1), while HR shows similar values during the test (Figure 2).

**Figure 3.**
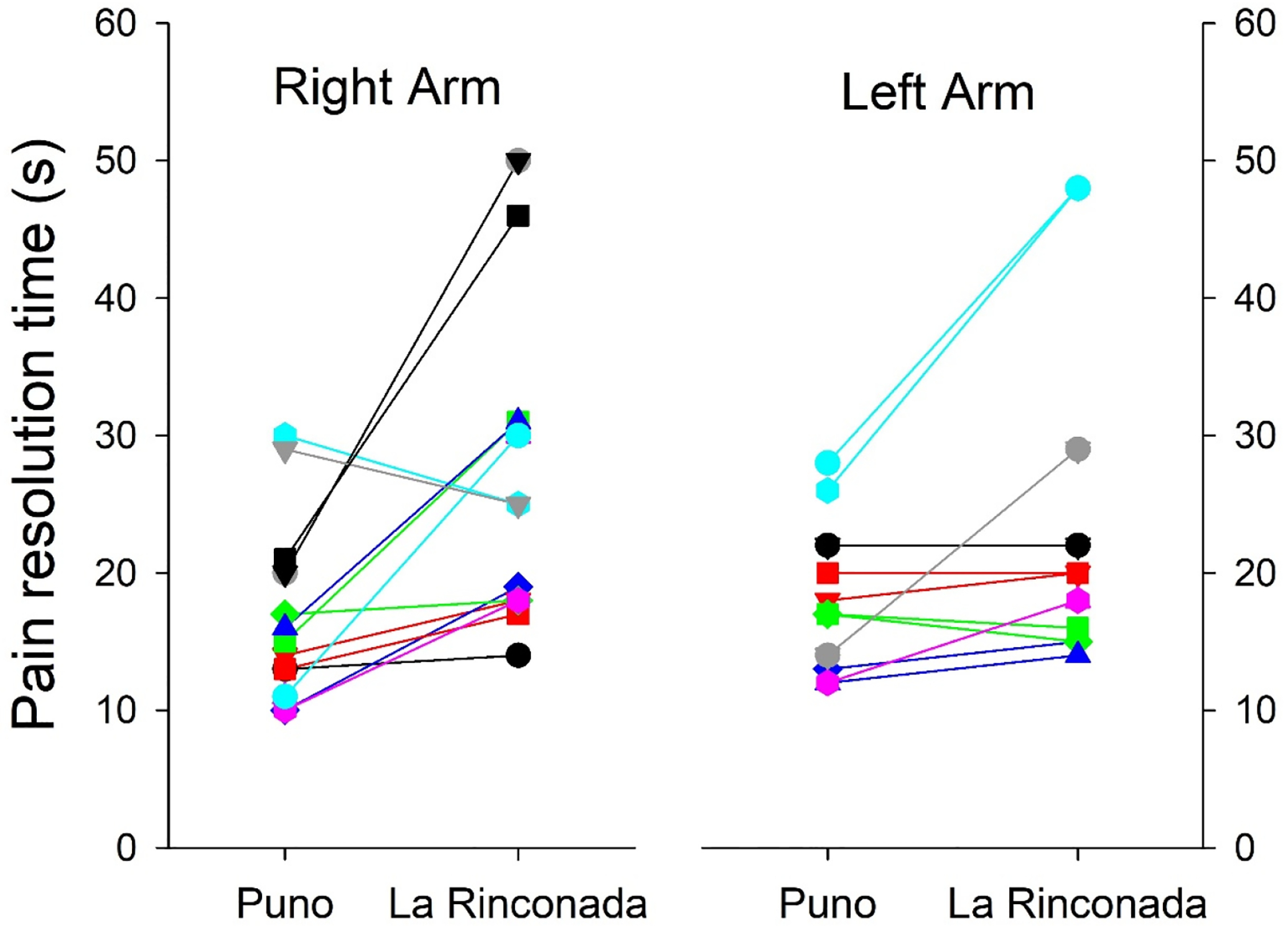
Individual changes in pain resolution time between the two altitudes in both arms.

**Figure 4.**
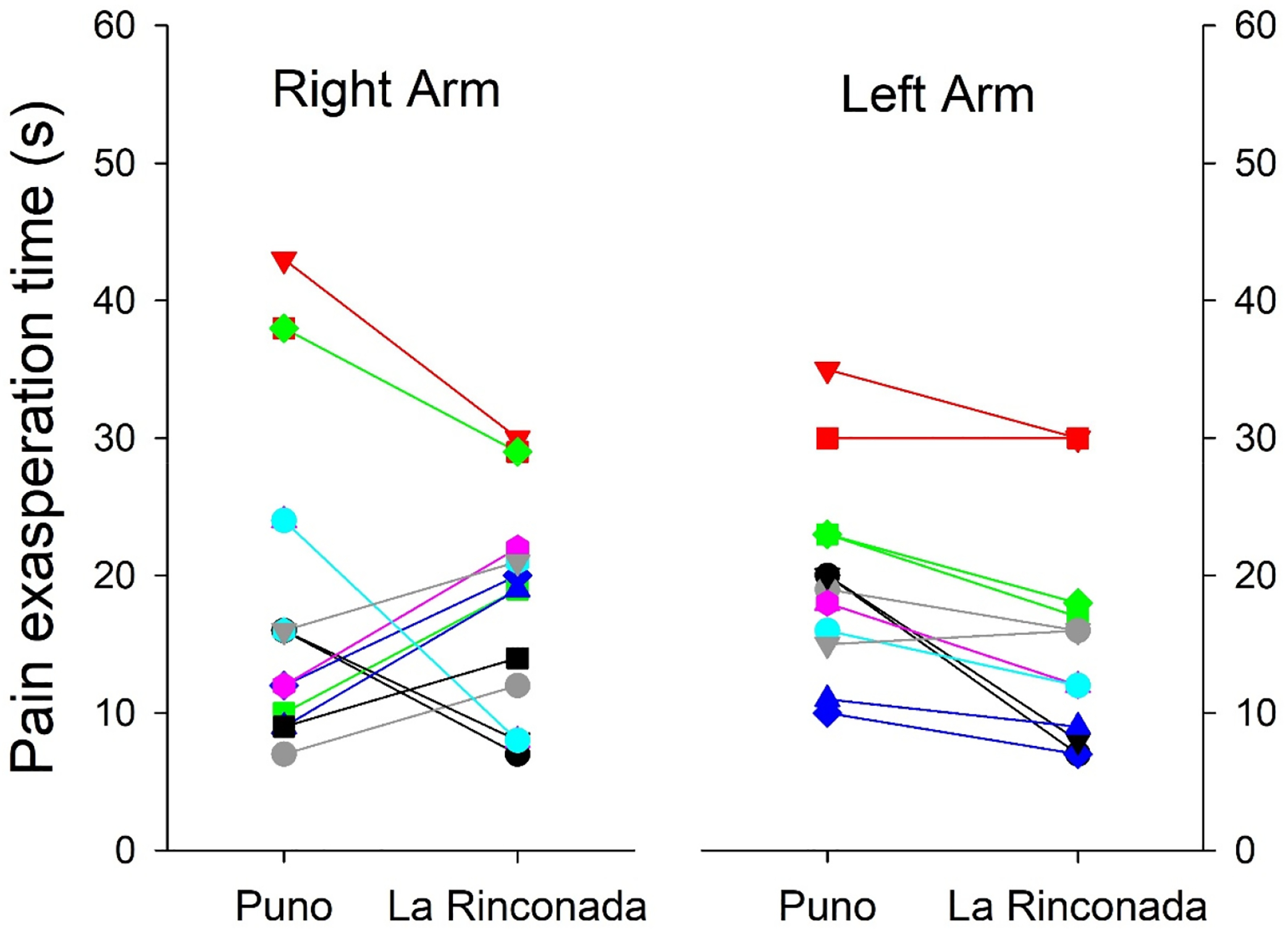
Individual changes in pain exasperation time between the two altitudes in both arms.

It is known that the ability to perceive pain presents differences associated with gender; for that reason, we disaggregated the data for men and women. Apart from the already-known differences between genders for hematological parameters and peripheral arterial saturation [15], we also observed that both SBP and DBP were slightly higher in men at both altitudes (Table 4). Minimal differences were observed in the time of exasperation at both altitudes concerning gender; in both cases, the difference was minimal, as it was for the resolution time of pain (Table 5). However, we must mention that a bimodal behavior is observed; the response both in exasperation time and in resolution time is not uniform, thus indicating the existence of at least two marked patterns in the individual differences in pain tolerance (Figures 3 and 4). Minimal differences were observed in pain perception at both altitudes, such as an earlier delivery in the left arm at both altitudes, a shorter excruciating pain in women at 3800 m, and a longer duration in women at 5100 m (Table 6). Minimal differences were also observed in the exasperation time and the resolution time at both altitudes.

## 4. Discussion

Pain is an unpleasant sensation where perception depends on the degree of injury and the speed of neuronal conduction [2]. Approximately 50% of patients go to the doctor’s office presenting some type of pain, with chronic pain having a prevalence between 20% and 40% [16], thus occasioning a serious impact on their quality of life. An adequate and holistic assessment of pain is often not part of the clinical routine assessment. In addition, the intensity and capacity of perception can vary depending on environmental, social, and genetic factors [17]. Among the environmental factors that could influence pain perception are environmental temperature, humidity, barometric pressure, and wind. An additional factor that can heavily influence pain perception is hypoxia, since the lower availability of oxygen could alter pain perception [18] through mechanisms that are yet to better understood.

The objective of the study was to evaluate whether or not there are changes in the sensory perception of pain when young, healthy high-altitude natives are exposed to an acute hypoxic environment. Baseline measurements were first made at their place of residence and, subsequently, at 5100 m after 48 h of staying in this environment. An increase was observed in SBP, DBP, Hb, and Hct, while there was a decrease in SatO_2_ and HR (Table 1). The slight changes observed in these variables are probably due to the acclimatization to the severe altitude of La Rinconada [19]. No other changes affecting the health conditions of the individuals studied were reported. No differences have been observed concerning gender, as both men and women presented similar findings (Tables 5 and 6). These results differ from a previous study that found differences in pain tolerance associated with gender indicating that women have a better tolerance [17].

The time elapsed until the appearance of the first pain sensation was slightly greater at 3800 than at 5100 m (Tables 2 and 3); however, these parameters were not statistically significant, probably because of the bimodal behavior of the sample, as can be observed in Figures 3 and 4. This bimodal model was also reflected in the exasperation time and the resolution time. Since this pattern is observed in both sexes, gender has no influence; consequently, differences could be due to genetic or psychological factors. Previous reports have suggested that pain perception differs mainly due to genetic factors, which could cause this difference [20]. We have found that at higher altitude, the painful sensation seems to be slightly delayed, but the exasperation and pain resolution times were slightly shorter at 5100 m compared to 3800 m. This suggests that the hypoxic environment could be conducive to a greater capacity to perceive pain. These findings coincide with previous studies, which showed a decrease in pain threshold by up to 40% [7]. In this report, it was also observed that the increase in pain perception is also related to other stimuli, such as the decrease in touch and smell sensation after the administration of oxygen. Nonetheless, our study is supportive of the hypothesis that hypoxia can alter pain perception. Mechanistically, this could be due to oxygen bioavailability and subsequent differences in nerve impulse conduction velocity when a subject is exposed to a hypoxic environment. This supposition requires further experimental confirmation. Also, the individual difference between both arms in the evolution of heart rate and arterial saturation during the tests is striking (Figures 3 and 4) without this behavior being attributable to gender or laterality in dexterity.

Changes in SatO_2_ and HR during the procedure have been evaluated (Table 4), showing an increase during the test compared to baseline conditions. This increase was progressive and in the same proportion, and is probably due to pain stimulating an increase in the respiratory rate. The proportional changes in SatO_2_ and HR during the procedure were lower at 5100 m compared to 3800 m and might be related to the lower barometric pressure [17]. Hypobaric hypoxia may coincide with the different phases of pain stimulation by the cuff pressure as it relates to breathing. Similar relations were observed with heart rate, where a progressive decrease was observed with the lowest value observed at the end of the test. These results are contrary to the physiological changes inherent to the painful stimulus that would normally increase heart rate [17]. These results are also inconsistent with reports of a lower rate of neuronal transmission that would allow the delayed transmission of painful sensations to the autonomic system without immediate changes in vital signs [2]. It is necessary to mention that the pulse oximeter was placed on a finger of the hand that was not involved during the procedure, preventing the pressure exerted by the cuff from generating these changes.

One of the mechanisms for better pain tolerance in acute hypoxia is related to the changes in neurotransmitters. An increase in catecholamines could help improve tolerance to pain since previous studies showed an increase in catecholamines of up to 36% during exposure to an altitude of 4300 m [20]. An increase of up to 99% in the arterial concentration of adrenaline was also determined during acute exposure, which subsequently decreases [21]. The increase in these neurotransmitters could generate a greater tolerance to pain, as has been observed in this study. Additional mechanisms could be related to the cardiovascular system and induced by the autonomic nervous system. Previous studies reported tachycardia at high altitudes [22]. However, we have observed a slight decrease in basal HR at the higher altitude, a finding that does not coincide with the results obtained in these studies, which may be due, at least to some extent, to the fact that the subjects were all natives of a high-altitude environment. Tachycardia is due to a muscarinic effect. Thus, a muscarinic blockade at rest and during physical exercise could reduce HR. In our study, the decrease in HR could be explained by a previous basal adaption to a moderate hypoxic environment prior to the test. It is also known that residents of high-altitude regions have hyposensitivity to pain in the cardiovascular system in hypoxia and hypersensitivity to parasympathetic stimulation, allowing the subjects to have a decrease in HR [23–25].

In this study, the accuracy of time measurement in seconds was a limitation that may have prevented the detection of slight differences. Although many parameters did not reach statistical significance, our data indicate the need for a larger sample size and additional complementary approaches. Furthermore, assessing pain tolerance in this way is straightforward and does not require expensive, sophisticated instruments. It can be performed by trained health personnel and is easy to interpret, making it a potentially useful tool. While this method may not be ideal for clinical practice, it could be valuable for evaluating apparent pain tolerance in primary care. Despite the limitations, we observed that a severely hypoxic environment can potentially influence nervous pain transmission since the nervous system is highly dependent on oxygen availability [24]. We also emphasize the importance of carrying out similar studies on subjects living in a severely hypoxic environment compared to subjects that live permanently at sea level, as the magnitude of pain perception could be different between sea-level and high-altitude environments.

## 5. Conclusions

The pain threshold seems to be slightly higher during exposure to a hypoxic environment; however, no statistical significance was observed. In addition, no differences in pain threshold were observed between men and women. Although there was no statistically significant difference, we observed subtle changes in the ischemic pain threshold between altitudes, which points to certain differences associated with exposure to acute hypoxia in young healthy Andean plateau natives when exposed to even higher altitudes.

## 6. Limitations

The Puno residents may have already been pre-conditioned to a moderate hypoxic environment before the study.

## Figures and Tables

**Table 1.** Baseline parameters and vital signs at 3800 m and 5100 m. Mean values ± standard deviation.

	Puno (3828 m)	La Rinconada (5100 m)	*p*	Cohen’s d
Sistolic Blood Pressure (mmHg)	105.29 ± 7.70	116.00 ± 4.77	0.000	−0.021
Diastolic Blood Pressure (mmHg)	70.79 ± 11.45	79.43 ± 2.34	0.008	−0.021
Hemoglobin (g/dL)	16.16 ± 2.29	17.57 ± 1.74	0.038	−0.032
Hematocrit (%)	48.79 ± 7.03	53.00 ± 5.33	0.034	−0.032
Oxygen saturation (%)	89.93 ± 2.01	84.29 ± 2.19	0.000	−0.021
Heart Rate (bpm)	95.21 ± 11.67	87.57 ± 9.39	0.062	−0.021

**Table 2.** Heart rate, peripheral oxygen saturation, and time course of pain during the test in the right arm.

	RIGHT ARM				
		Puno (3828 m)	La Rinconada (5100 m)	Cohen’s d	*p*
Time (s)	First pain	30.43 ± 14.15	31.00 ± 19.01	−0.032	0.941
	Unbearable pain	48.43 ± 16.00	48.00 ± 19.45	−0.10	0.955
	No pain	64.86 ± 18.01	74.57 ± 19.87	−0.05	0.286
Oxygen Saturation (%)	First pain	88.86 ± 3.65	84.29 ± 2.09	−0.54	0.004
	Unbearable pain	89.29 ± 2.61	85.21 ± 1.53	−0.48	0.000
	No Pain	90.57 ± 2.59	86.86 ± 1.87	−0.32	0.001
Heart Rate (bpm)	First pain	95.43± 16.95	87.36 ± 9.02	−0.26	0.135
	Unbearable pain	91.43 ± 13.43	84.07 ± 6.47	−0.32	0.052
	No Pain	83.43 ± 15.97	80.00 ± 11.52	−0.43	0.542

**Table 3.** Heart rate, peripheral oxygen saturation, and time course of pain during the test in the left arm.

	LEFT ARM				
		Puno (3828 m)	La Rinconada (5100 m)	Cohen’s d	*p*
Time (s)	First pain	19.93 ± 9.44	23.07 ± 10.83	−0.32	0.114
	Unbearable pain	39.50 ± 7.10	37.79 ± 10.39	−0.05	0.475
	No pain	57.14 ± 11.24	61.64 ± 14.81	−0.32	0.111
Oxygen Saturation (%)	First pain	89.21 ± 2.99	83.29 ± 4.19	−0.43	0.001
	Unbearable pain	89.64 ± 3.03	83.00 ± 2.60	−0.43	0.000
	No Pain	91.86 ± 2.56	85.21 ± 3.55	−0.37	0.000
Heart Rate (bpm)	First pain	90.86 ± 12.83	92.50 ± 12.46	0.52	0.702
	Unbearable pain	92.21 ± 15.16	95.14 ± 9.40	−0.32	0.533
	No Pain	79.79 ± 12.54	82.71 ± 5.39	0.42	0.475

**Table 4.** Baseline parameters of vital signs at 3800 m and 5100 m by gender.

	Puno (3828 m)			La Rinconada (5100 m)		
	Men	Women	*p*	Men	Women	*p*
Sistolic Blood Pressure (mmHg)	109.00 ± 6.45	102.50 ± 7.73	0.122	117. 67 ± 0.52	114.75 ± 6.16	0.274
Diastolic Blood Pressure (mmHg)	76.67 ± 10.80	66. 38 ± 10.41	0.097	80.01 ± 2.36	79. 00 ± 2.39	0.452
Hemoglobin (g/dl)	18.36 ± 1.34	14.51 ± 1.09	0.000	18.67 ± 1.56	16.75 ± 1.46	0.036
Hematocrit (%)	55.50 ± 4.13	43.75 ± 3.45	0.000	56.33 ± 4.67	50.50 ± 4.53	0.037
Oxygen saturation (%)	89. 67 ± 2.16	90.13 ± 2.03	0.691	84.00 ± 0.89	84.50 ± 2.88	0.691
Heart Rate (bpm)	97.33 ± 7.20	93.63 ± 14.46	0.577	89.00 ± 11.63	86.50 ± 8.02	0.641

**Table 5.** Pain perception changes by gender during the test in the right arm.

		RIGHT ARM					
		Puno (3828 m)			La Rinconada (5100 m)		
		Women	Men	*p*	Women	Men	*p*
Time (s)	First pain	32.00 ± 17.33	28.33 ± 9.56	0.65	30.75 ± 22.79	31.33 ± 14.56	0.96
	Unbearable pain	49.50 ± 20.92	47.00 ± 6.99	0.78	51.38 ± 23.19	43.50 ± 13.69	0.48
	No pain	64.88 ± 20.48	64.83 ± 16.02	0.99	80.25 ± 18.79	67.00 ± 20.29	0.23
	Exasperation time	17.50 ± 14.35	18.67 ± 4.13	0.85	20.63 ± 6.37	12.17 ± 6.85	0.04
	Pain resolution time	15.38 ± 4.07	17.83 ± 9.88	0.51	28.88 ± 13.09	23.50 ± 6.56	0.37

**Table 6.** Pain perception changes by gender during the test in the left arm.

		LEFT ARM					
		Puno (3828 m)			La Rinconada (5100 m)		
		Women	Men	*p*	Women	Men	*p*
Time (s)	First pain	16.38 ± 7.93	24.67 ± 9.83	0.11	22.88 ± 13.58	23.33 ± 6.83	0.94
	Unbearable pain	37.13 ± 5.59	42.67 ± 8.14	0.16	40.75 ± 10.89	33.83 ± 9.06	0.23
	No pain	52.75 ± 7.23	63.00 ± 13.54	0.09	60.50 ± 8.93	63.17 ± 21.31	0.75
	Exasperation time	20.75 ± 8.83	18.00 ± 1.79	0.47	17.88 ± 8.44	10.50 ± 2.35	0.06
	Pain resolution time	15.63 ± 2.77	20.33 ± 6.86	0.10	19.75 ± 6.14	29.33 ± 14.57	0.12

## Data Availability

Data is contained within the article.
